# Supplementary material for: Causal Relationship between Aging and Anorexia Nervosa: A White-Matter-Microstructure-Mediated Mendelian Randomization Analysis
Source: Biomedicines. 2024 Aug 16;12(8):1874. doi: 10.3390/biomedicines12081874 (PMC11351342; doi:10.3390/biomedicines12081874)
Supplement: Supplementary file 1 [file biomedicines-12-01874-s001.zip › Supplementary Figure/Figure S4/Singe SNP plot of MR analysis for TL to ubm-b-1722.pdf]

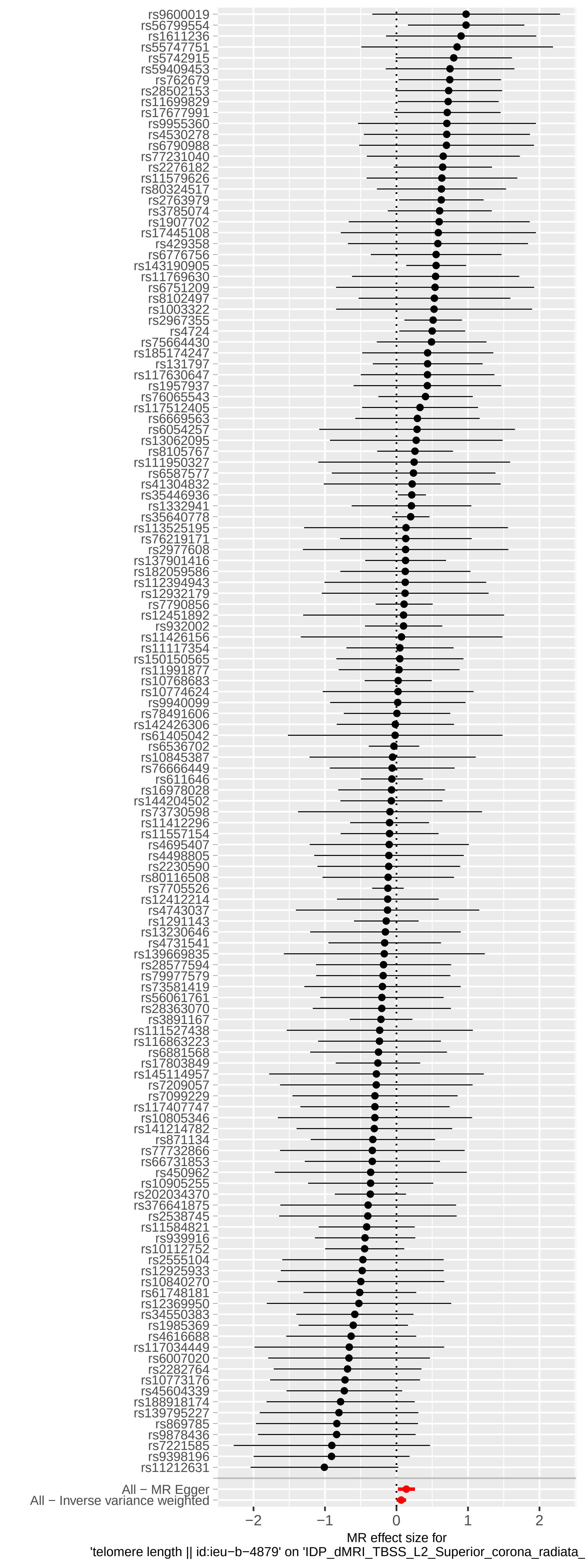
rs9600019
rs56799554
rs1611236
rs55747751
rs5742915
rs59409453
rs762679
rs28502153
rs11699829
rs17677991
rs9955360
rs4530278
rs6790988
rs77231040
rs2276182
rs11579626
rs80324517
rs2763979
rs3785074
rs1907702
rs17445108
rs429358
rs6776756
rs143190905
rs11769630
rs6751209
rs8102497
rs1003322
rs2967355
rs4724
rs75664430
rs185174247
rs131797
rs117630647
rs1957937
rs76065543
rs117512405
rs6669563
rs6054257
rs13062095
rs8105767
rs111950327
rs6587577
rs41304832
rs35446936
rs1332941
rs35640778
rs113525195
rs76219171
rs2977608
rs137901416
rs182059586
rs112394943
rs12932179
rs7790856
rs12451892
rs932002
rs11426156
rs11117354
rs150150565
rs11991877
rs10768683
rs10774624
rs9940099
rs78491606
rs142426306
rs61405042
rs6536702
rs10845387
rs76666449
rs611646
rs16978028
rs144204502
rs73730598
rs11412296
rs11557154
rs4695407
rs4498805
rs2230590
rs80116508
rs7705526
rs12412214
rs4743037
rs1291143
rs13230646
rs4731541
rs139669835
rs28577594
rs79977579
rs73581419
rs56061761
rs28363070
rs3891167
rs111527438
rs116863223
rs6881568
rs17803849
rs145114957
rs7209057
rs7099229
rs117407747
rs10805346
rs141214782
rs871134
rs77732866
rs66731853
rs450962
rs10905255
rs202034370
rs376641875
rs2538745
rs11584821
rs939916
rs10112752
rs2555104
rs12925933
rs10840270
rs61748181
rs12369950
rs34550383
rs1985369
rs4616688
rs117034449
rs6007020
rs2282764
rs10773176
rs45604339
rs188918174
rs139795227
rs869785
rs9878436
rs7221585
rs9398196
rs11212631
All − MR Egger
All − Inverse variance weighted
−2
−1
0
1
2
MR effect size for
'telomere length || id:ieu−b−4879' on 'IDP_dMRI_TBSS_L2_Superior_corona_radiata_
